# Summer Heat and Mortality in New York City: How Hot Is Too Hot?

**DOI:** 10.1289/ehp.0900906

**Published:** 2009-09-10

**Authors:** Kristina B. Metzger, Kazuhiko Ito, Thomas D. Matte

**Affiliations:** 1 Bureau of Environmental Surveillance and Policy, New York City Department of Health and Mental Hygiene, New York, New York, USA;; 2 Department of Environmental Medicine, New York University School of Medicine, New York, New York, USA

**Keywords:** epidemiology, heat wave, meteorology, mortality, temperature

## Abstract

**Background:**

To assess the public health risk of heat waves and to set criteria for alerts for excessive heat, various meteorologic metrics and models are used in different jurisdictions, generally without systematic comparisons of alternatives. We report such an analysis for New York City that compared maximum heat index with alternative metrics in models to predict daily variation in warm-season natural-cause mortality from 1997 through 2006.

**Materials and methods:**

We used Poisson time-series generalized linear models and generalized additive models to estimate weather–mortality relationships using various metrics, lag and averaging times, and functional forms and compared model fit.

**Results:**

A model that included cubic functions of maximum heat index on the same and each of the previous 3 days provided the best fit, better than models using maximum, minimum, or average temperature, or spatial synoptic classification (SSC) of weather type. We found that goodness of fit and maximum heat index–mortality functions were similar using parametric and nonparametric models. Same-day maximum heat index was linearly related to mortality risk across its range. The slopes at lags of 1, 2, and 3 days were flat across moderate values but increased sharply between maximum heat index of 95°F and 100°F (35–38°C). SSC or other meteorologic variables added to the maximum heat index model moderately improved goodness of fit, with slightly attenuated maximum heat index–mortality functions.

**Conclusions:**

In New York City, maximum heat index performed similarly to alternative and more complex metrics in estimating mortality risk during hot weather. The linear relationship supports issuing heat alerts in New York City when the heat index is forecast to exceed approximately 95–100°F. Periodic city-specific analyses using recent data are recommended to evaluate public health risks from extreme heat.

It has long been known that elevated summertime temperatures are associated with increased daily mortality (Basu and Samet 2002). This relationship is most dramatic during heat waves (several days of extreme heat) with the occurrence of heat stroke deaths [(Centers for Disease Control and Prevention (CDC) 2005; New York City Department of Health and Mental Hygiene (NYCDOHMH) 2006; Semenza et al. 1996] and excess natural-cause mortality (Davis et al. 2003). Many communities issue heat advisories before these periods of excessive heat to provide advance warning of dangerously hot weather and to allow for a timely response by local agencies.

Most cities in the United States use National Weather Service (NWS) excessive heat alerts (advisories, watches, and warnings) based on the 24- to 48-hr forecast maximum heat index, a metric intended to reflect perceived temperature based on the measured temperature and relative humidity. An advisory is issued when the heat index is forecast to exceed 100°F (35°C) and 105°F (38°C) for northern and southern locations, respectively (NWS 2008a). Increasingly, other cities in the United States, such as Philadelphia (Kalkstein et al. 1996), and at least two dozen other cities worldwide (Sheridan and Kalkstein 2004), are using the Heat Health Watch and Warning System (HHWWS) as an alternative for heat alerts. The HHWWS uses daily forecasts of the dominant local weather pattern—the spatial synoptic classification (SSC)—to calculate predicted excess deaths based on location-specific historical mortality data (Sheridan 2002).

One criticism of using heat index–based alerts, rather than a HHWWS, is that the criteria are not specific for the health risks of the local population, particularly in urban areas (Tew et al. 2004). The differences in the heat–mortality relationship between communities, with northern cities experiencing a greater mortality impact from higher summertime temperatures than southern cities (Curriero et al. 2002; McMichael et al. 2008), may be more nuanced than the difference in latitude. For example, the urban heat-island effect causes urban centers to experience higher daytime and nighttime temperatures than surrounding areas (Gaffin et al. 2008), and microclimate effects within cities (Harlan et al. 2006) may also play a role. These factors make some populations in urban areas more vulnerable to heat-related morbidity and mortality (Buechley et al. 1972; Smoyer 1998). Regardless of the weather metric used to model risk, the use of mortality data from previous decades (Ebi et al. 2004) may not accurately reflect current risk because of declining overall mortality (Kung et al. 2008) and changes in population vulnerability to heat (Carson et al. 2006; McMichael et al. 2006).

As part of an effort to evaluate the New York City criteria for issuing heat advisories (NWS 2008b) and activating emergency responses to extreme heat, we evaluated heat index, temperature, SSC categories, and additional meteorologic factors as predictors of natural-cause mortality during summertime weather conditions. The analyses used 10 years of daily mortality data on New York City residents for the months of May through September from 1997 through 2006 and compared the goodness of fit among models with various parametric and nonparametric functions and lag structures relating weather to mortality.

## Materials and Methods

### Meteorologic data

Hourly meteorologic data were obtained from the National Climatic Data Center for the three New York City stations located at Central Park, LaGuardia Airport, and John F. Kennedy Airport for the period 1997 through 2006 (National Climate Data Center 2007). Daily summaries were created from the hourly measurements at each station. After assessing the relation among data from the three weather stations, we chose to use meteorologic data from LaGuardia Airport because it had the most complete records during the study period (95% of hours, 100% of days with at least 14 hr of data). Because the NWS in the United States uses the Fahrenheit scale for conventional heat advisory, watch, and warning criteria, we used it to report all meteorology metrics. During the warm season, temperatures at LaGuardia tended to be slightly higher than at the other two weather stations (with maximum temperature on average 1°F and 2°F warmer than Central Park and John F. Kennedy Airport, respectively); maximum temperature was highly correlated among the three weather stations (*R* > 0.93).

The weather metrics we evaluated included minimum, maximum, and average (mean of minimum and maximum) temperature (°F), sea level pressure (mbar), wind speed (miles per hour), and precipitation (any vs. none). An exponential function of temperature above a threshold {0.1 × exp [0.2 × (T_max_ – 90)]} where T_max_ is maximum temperature was also considered given its previous use in examining the relationship of temperature and mortality in New York City (Buechley et al. 1972; Marmor 1975). Heat index was calculated using ambient temperature (°F) and relative humidity (%) for ambient temperature of ≥ 80°F and relative humidity of ≥ 40% (Steadman 1979). For this analysis, the daily maximum heat index is the larger value of maximum temperature or maximum heat index on each day, which was the best measure of the highest perceived daily temperature. Daily deviation from maximum temperature was calculated using as a reference period the average maximum temperature at LaGuardia Airport from 1971 through 2000 (NWS 2009).

Daily spatial synoptic classification (SSC) data were obtained for LaGuardia Airport during the same time period (Sheridan 2008). SSC is a classification system that uses temperature, dew point, wind direction, wind speed, and cloud cover measured four times daily to categorize surface weather conditions on each day into six main types and two subtypes (Sheridan 2002). Our analyses focused on seven weather types that occurred during the warm season in New York City: dry polar, dry moderate, dry tropical, moist polar, moist moderate, moist tropical, moist tropical plus, and transitions.

### Mortality data

Data on every death of a New York City resident that occurred in the five boroughs (The Bronx, Brooklyn, Manhattan, Queens, and Staten Island) for the period 1997 through 2006 were obtained from the NYCDOHMH Office of Vital Statistics. Data included underlying and contributing cause of death, age, sex, race, place of birth, place of death, and census tract of residence. The NYCDOHMH Institutional Review Board approved this study.

To classify cause of death, we used the *International Classification of Diseases, 10th revision* (ICD-10; WHO 2007) for the 1999 through 2006 data; the ICD-9 (WHO 2009) was used for the years 1997 to 1998. The outcome of interest was natural-cause mortality, which included deaths with underlying cause of death exclusive of external causes (ICD-10, A00–R99; ICD-9, 001–799).

### Analytic methods

We developed Poisson time-series regression models to relate daily natural-cause mortality to daily weather conditions during the warm season (May through September). Because the focus of the study was heat-related mortality, the seasonal restriction simplified the control for seasonal trends by eliminating the larger wintertime mortality variation associated, in part, with influenza and other communicable diseases (Hajat et al. 2002).

To assess the robustness of our findings, two types of analytic models—parametric generalized linear models and nonparametric generalized additive models were analyzed in parallel. Using parametric models, we investigated models with deterministic functions (i.e., linear, quadratic, cubic, sine, and cosine) and their interactions with year. The parametric models were developed using PROC GENMOD (version 9.1.3; SAS Institute Inc., Cary, NC). Models with nonparametric smoothing terms were developed to estimate the shape of potential nonlinear relationships between temperature and mortality and to compare them with the functional forms obtained from parametric models. In the nonparametric models, the degree of smoothness of model terms was estimated as part of fitting. Penalized splines were used to assess the optimum effective degrees of freedom for temporal and meteorologic terms (Wood 2004), as implemented in R statistical software (version 2.7.1; R Core Development Group, Vienna, Austria).

Our model-building process involved first adjusting for long- and short-term temporal trends only. In parametric models, to control for long-term and seasonal temporal trends, we included linear and quadratic terms for the day of year and their interaction terms with year, plus sine and cosine terms (1 period per year) to control for seasonality. In nonparametric models, the number of degrees of freedom used to control for long-term and seasonal temporal trends was based on the number that yielded the minimum of the sum of the absolute values of partial autocorrelation. This method allows control for a mixture of unmeasured confounders, such as infectious diseases and influenza epidemics, without penalizing for the amount of “wiggle” in the fitted temporal trend. Such methods have been applied to adjust for temporal trends in estimating air pollution effects (Peng et al. 2006; Touloumi et al. 2006). We tested models using between 2 and 10 degrees of freedom per season and found that 5 degrees of freedom per season gave the optimum result. Indicator variables for day of week, holidays, the World Trade Center attacks (11 September 2001), and the Northeast blackout (15 August 2003) were added to control for short-term temporal trends. The fitted series of the temporal nonparametric model was similar to that the temporal parametric model.

Next, we developed meteorology models, built upon the base temporal models, for each candidate meteorologic metric intended to reflect daily population heat exposure. The following metrics were assessed: minimum, average, and maximum temperature, maximum heat index, the exponential temperature transformation, SSC (categorical), and deviation from normal maximum temperature (categorical). For each metric, lagged functions of up to 1 week were assessed to identify those that improved model fit, with the constraint of including all intermediate lags of the shortest and longest lags deemed important (Armstrong 2006). We also evaluated the average of multiday lags but found that the models with multiple single-day lags often resulted in at least as good a fit as the multiday averages or combinations of single days and the average of multiday lags. For example, a model with lags of 0 through 3 days from the maximum heat index yielded approximately the same deviance explained as a model with the same day and the average of 1-, 2-, and 3-day lags. Thus, models with single-day lags were preferred to allow interpretation of results for each day and the ability to estimate excess mortality risk for consecutive hot days. In parametric models, linear, quadratic, and cubic terms for each continuous meteorologic variable were included together to allow for a nonlinear relationship of temperature and mortality (Armstrong 2006). In nonparametric models, penalized splines were used to simultaneously fit each of the individual lagged meteorologic variables. This resulted in degrees of freedom of between 1 and approximately 8 over the variable range, depending on the lag. Thus, although the nonlinear shape of the temperature–mortality relationships estimated at each lag day could be unstable when multiple-day lags were included simultaneously (as with the instability of individual estimates in an unconstrained distributed lag model), the results suggest consistency of the shape estimated by these two models.

We compared single-metric meteorology models for fit and explanatory ability (Armstrong 2006). Model fit was evaluated using percent deviance explained, first-order residual autocorrelation, and correlation of raw and predicted values on days with heat index ≥ 90°F (to assess the model fit during hotter weather).

In sensitivity analyses, we evaluated how much the fit of each model based on a single heat-related meteorologic metric was improved by adding additional meteorologic variables, including average sea level pressure (linear), average wind speed (linear), and precipitation (any precipitation, no precipitation), as well as SSC categories.

We also assessed the potential impact of seasonal acclimatization on mortality. We directly examined potential effect modification of the relation of temperature and mortality by testing interaction terms with day of year and month. Indirect assessment was conducted using the model with deviation from normal maximum temperature, because a given maximum temperature represents a greater deviation from normal when it occurs earlier in the season.

## Results

On average, 136 New Yorkers died from natural causes each day during the warm season (May through September) from 1997 through 2006 (Table 1). For the 10-year study period, a gradual downward trend was observed in the average number of daily natural-cause deaths (144 in 1997 to 129 in 2006). In the warm season, average daily mortality was highest at the beginning of May and reached its nadir in mid-August.

During the study period, the average daily temperature at LaGuardia Airport was 71°F, the average maximum temperature was 80°F, and the average maximum heat index was 80°F in the warm season (Table 1). Of the 5 months, July (average daily temperature, 77°F; average maximum heat index, 87°F) and August (average daily temperature, 76°F; average maximum heat index, 86°F) were the warmest months. Temperatures peaked at the end of July.

The different meteorologic metrics that represent daily population heat exposure were moderately to strongly correlated (Table 1). Maximum temperature and maximum heat index were very strongly correlated (*R* = 0.986). Average barometric pressure and average wind speed were negatively correlated with temperature metrics, but the relation was weak.

During the warm season, 7% of days were included in the SSC category dry tropical, 22% were moist tropical, and 8% were moist tropical plus. The proportion of days classified into each of these categories increased as the maximum heat index increased. At heat indices of ≥ 100°F (*n* = 39), dry tropical and moist tropical plus described 44% and 49% of days, respectively. The mean maximum heat index was 91°F on dry tropical days, 85°F on moist tropical days, and 91°F on moist tropical plus days.

The parametric model with a single meteorologic factor that had the best overall fit considering all three criteria—greatest proportion of deviance explained, smallest first-order autocorrelation, and largest correlation of raw and predicted values on hot days—included linear, quadratic, and cubic terms for maximum heat index on the same day and previous 3 days (lags 0–3; Table 2). This model fit the data moderately better than did the four other models with a single meteorologic metric, including cubic functions of maximum temperature (lags 0–3), minimum temperature (lags 0–3), average temperature, and SSC (lags 0–3; Table 2). The models with the exponential temperature transformation (lags 0–1) and categories of deviation of normal maximum temperature (lags 0–1) fit the data least well among these single meteorologic metric models. However, the performance difference between the best- and worst-fitting model was not large as measured by the proportion of daily mortality variation (i.e., deviance) explained: 27.9% for the SSC model and 24.7% for the deviation from maximum temperature model.

Results of nonparametric models were similar (Table 2). The models with maximum heat index or with average, minimum, or maximum temperature on the same day and previous 3 days (lags 0–3) performed equally well. The model that used penalized splines to control for maximum heat index suggested 1 degree of freedom (i.e., linear) for the same day and approximately 5, 8, and 7 degrees of freedom for lags of 1, 2, and 3 days, respectively.

**Figure 1:**
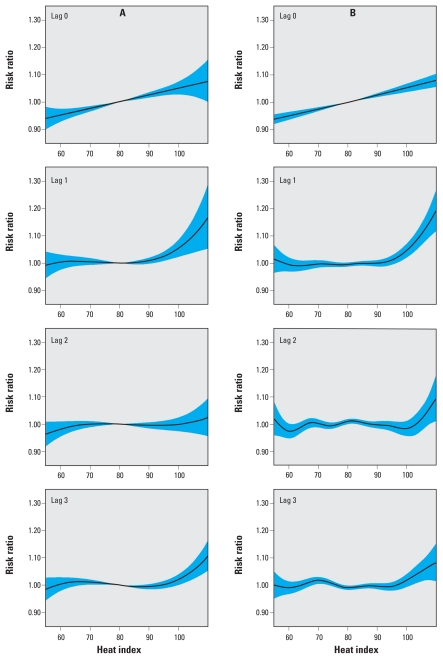
Risk ratios (and 95% CIs) for the association of daily natural-cause mortality by maximum heat index on the same day (lag 0) and 3 previous days (lags 1–3, respectively) using parametric generalized linear models (*A*) and nonparametric generalized additive models (*B*), New York City, 1997 through 2006. Each risk ratio function is adjusted for temporal covariates for year, season, and day of week and for the other lagged heat index terms.

**Figure 2:**
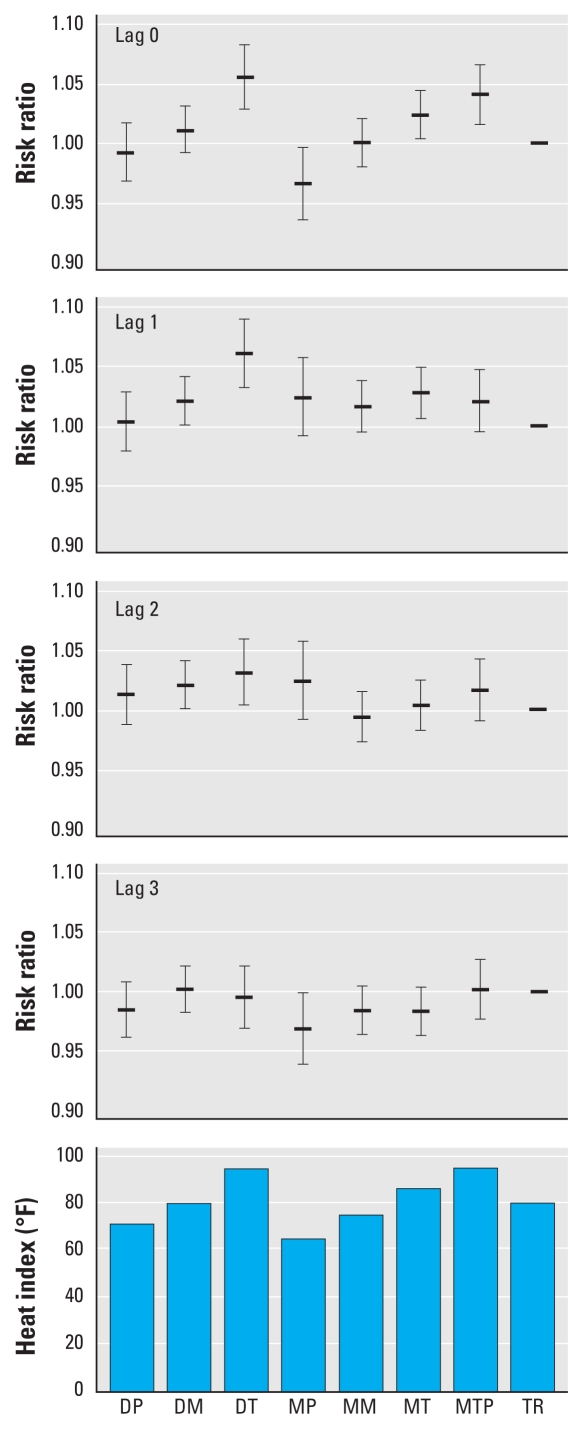
Risk ratios (and 95% CIs) for association of daily natural-cause mortality with spatial synoptic classification (SSC) categories (lags 0–3), and average daily maximum heat index by SSC on the same day (lag 0), New York City, 1997 through 2006. Each risk ratio function is adjusted for temporal covariates for year, season, and day of week. SSC categories: DP, dry polar; DM, dry moderate; DT, dry tropical; MP, moist polar; MM, moist moderate; MT, moist tropical; MTP, moist tropical plus; TR, transitions (Sheridan 2008).

The fitted relationships of daily maximum heat index to mortality were similar for the parametric and nonparametric models (Figure 1). On the same day, the relationship appeared to be linear across the range of observed heat index. The relations of mortality to heat index on the 3 previous days, however, were relatively flat across a range of moderate seasonal temperatures but increased sharply at a heat index between approximately 95°F and 100°F. The estimated risk ratios for same-day SSC categories were consistent with the relationship in mean maximum heat index among SSC categories, whereas the lagged effects of SSC were less consistent (Figure 2).

**Figure 3:**
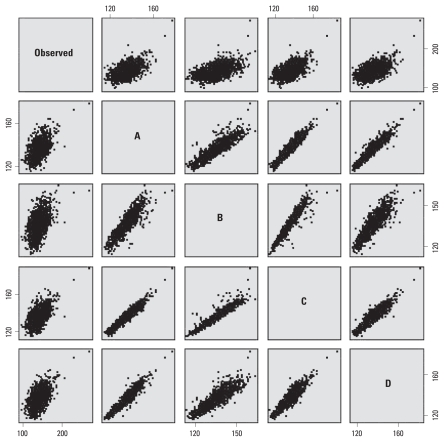
Scatterplot matrix of observed daily natural-cause mortality and predicted values from selected models where maximum heat index was ≥ 90°F. Both axes are daily counts. A through D indicate predicted values from the models that included alternative weather variables in addition to the terms for temporal trends: A, heat index only; B, SSC only; C, heat index plus SSC; D, heat index plus other meteorologic variables. For example, in the first row, second column of the plots, the *x*-axis indicates predicted values from model A and the *y*-axis indicates observed natural-cause mortality; in the second row, first column, the *x*-axis indicates the observed data and the *y*-axis indicates the predicted values from Model A. Each model included temporal covariates for year, season, and day of week.

In sensitivity analyses, we assessed how much the fit of the maximum heat index model could be improved with the addition of other meteorologic variables. The first sensitivity analysis added SSC categories (lags 0–3) to the final heat index model. The second added cubic terms for minimum temperature (lags 0–3), mean sea level pressure (lags 0–1), mean wind speed (lags 0–1), and precipitation (lags 0–3). Terms were added sequentially and determined to be statistically significant in the model. Each of these alternative models fit the data better than the heat index only model (Table 2). Although the risk ratios for heat index attenuated slightly in both models, the shapes of the curves remained similar. Additionally, the correlations of predicted values on hot days (heat index ≥ 90°F) among these models were high (Table 2, Figure 3).

We found little evidence for effect modification of temperature by time of year during this time period. Using the maximum heat index parametric model as the base model, all interaction terms between maximum heat index functions and within-season temporal trends, including day of year and month, were not significant. When stratifying the analysis by early warm season (May through July) to late warm season (August through September), the shape of the risk ratio response function did not differ significantly (data not shown). The results were essentially the same when we used data from 1990 through 2006 in a sensitivity analysis capture years with more hot weather in May.

## Discussion

In our analysis, several meteorologic metrics used to describe the extent of population heat exposure performed similarly as predictors of increased natural-cause mortality in New York City during the months of May to September from 1997 to 2006. We observed this association with same-day mortality across the range of seasonal temperatures, whereas days with maximum heat index of approximately 95–100°F or greater were associated with higher mortality on the following 3 days.

We found that using maximum heat index provided moderately better model fit in parametric generalized linear models compared with models using average, minimum, or maximum temperature (Hajat et al. 2002; Hajat et al. 2006; Huynen et al. 2001). Similar to a study assessing the impact of weather on the association of particulate matter and mortality, we found that models using smoothed functions of continuous temperature metrics have better fit than models using synoptic weather categories only (Samet et al. 1998). Additionally, the smoothed function approach, in both parametric and nonparametric models, described the relationship of mortality with successively higher heat indices more fully than simpler transformations of temperature (McMichael et al. 2008). It should be noted, however, that all of these models used highly correlated measures of temperature and heat index; the fit and predictive ability of the models were similar.

Our finding that the temperature–mortality relationship persisted for several days is consistent with previous research (Hajat et al. 2002; Hajat et al. 2006; Huynen et al. 2001). The magnitude of this lagged relationship increased sharply at heat indices between approximately 95°F and 100°F. Other studies of cities with climates similar to that of New York City have described an increase in mortality risk at temperatures within this range (Michelozzi et al. 2006). The lagged effect of extreme heat on mortality is also consistent with the observed lethality of heat waves with extremely high temperatures lasting several days (Tan et al. 2007).

We observed a nonlinear relationship of heat index and mortality at lagged days, but not on the same day in this data set. In contrast, two recent multicity studies found essentially linear relationships between temperature and mortality during the warm season (Anderson and Bell 2009; Zanobetti and Schwartz 2008). Both studies, however, limited their models to same-day temperatures (Zanobetti and Schwartz 2008) or 2-day averages of the same and previous day (Anderson and Bell 2009), for which we also found linear relationships in both parametric and nonparametric models. Those studies also noted that the temperature–mortality relationship differed by city, dependent on location and other community characteristics. Thus, although functional forms may vary from city to city, determining the shape of the temperature–mortality relationship needs to take into consideration lagged associations. If the temperature–mortality relationship were fully linear, weather criteria for heat alerts would be completely arbitrary. Our analysis of New York City data indicates that there is a linear component (same-day) and a nonlinear component (lagged) in the temperature–mortality relationship supporting issuing heat alerts when the heat index is forecast to exceed approximately 95°–100°F. At the same time, our analysis does not suggest a bright line below which no excess heat-related mortality occurs. Even if there was a statistical threshold for increased risk at the population level, individuals within the population may have different thresholds for experiencing life-threatening heat illness, depending on living conditions and health status.

Other studies have indicated that heat waves earlier in the season are more lethal (Hajat et al. 2002), possibly due to populations being less acclimatized to hot weather. Alternatively, if early heat waves result in “harvesting” or “mortality displacement,” the mortality impact of heat waves later in the summer is reduced among the now smaller pool of susceptible individuals. We found little evidence of a larger effect of early-season heat waves during our study period. There was no interaction between maximum heat index and month, nor did deviation from normal temperature fit the data as well as maximum heat index. This finding, however, could be a result of few unseasonably hot days occurring in May or June during this time period, although our sensitivity analysis including data from 1990–2006 was consistent with our main finding. Because of the infrequency of early-season heat waves, we do not feel our findings provide strong evidence either for or against a greater hazard of such events.

Our analysis of heat–mortality relationships used both parametric and nonparametric modeling approaches. The final models identified by each of these approaches were remarkably similar, yielding risk ratio functions of similar form and magnitude of effects. Although the large numbers of degrees of freedom used for smoothing temperature metrics in the nonparametric model seem more than that needed to describe a biologically plausible relationship between heat index and mortality (and likely reflect the year-to-year variation in the relationship), the overall shape of these terms does not deviate substantially from either linear, hockey-stick, or U-shaped curves. Nonparametric modeling has the flexibility to identify and account for trends in continuous variables that are more difficult to do with parametric functions. This flexibility, however, can enable overfitting of observed variation. The consistency of our findings using two different approaches suggests that we have estimated a relatively robust relationship between hot weather and mortality in New York City.

We focused our analyses on the warm season only in order to better describe the heat–mortality relationship, as several others have done (Vaneckova et al. 2008). Other studies have examined year-round weather and mortality data (Anderson and Bell 2009; Curriero et al. 2002; Huynen et al. 2001; McMichael et al. 2008). Factors that potentially confound the temperature–mortality relationship differ during the warm and cold seasons (Ito et al. 2007). In the winter, circulating influenza viruses tend to be the strongest correlates with daily mortality, whereas in the summer, the impact of communicable disease on mortality is negligible (CDC 2008; Mounts et al. 2000). Thus, an analysis limited to the warm season only may reduce the impact of seasonal mortality trends and increase the potential for model misspecification to conflate seasonal effects with short-term temperature effects.

The relationship of hot weather and population exposure to heat stress is complex, and other weather factors, such as precipitation and wind speed, likely play a role along with temperature and humidity. Individuals within a large and diverse city may experience different exposures to heat stress depending on their access to air conditioning (Bouchama et al. 2007), the characteristics of their dwelling (Semenza et al. 1996), and local microclimate effects (Harlan et al. 2006). Because of this complexity, a composite weather metric has the conceptual appeal of using all relevant weather data to assess the public health risks of hot weather. Our results showed that the fit of a model based on only maximum heat index was modestly improved by adding other weather metrics such as minimum temperature, barometric pressure, wind speed, and precipitation or the composite metric of SSC. Some of the added weather metrics are highly correlated with maximum heat index, however, and it is unclear that the resulting modest improvement in model fit justifies the use of a more complex model for risk assessment or guiding the issuance of heat advisories. For instance, the use of barometric pressure (either explicitly or through its association with SSC) in a weather–mortality model introduces an effect that may be mediated through mechanisms other than heat stress. For example, barometric pressure variation has been associated with risk of pulmonary embolism (Meral et al. 2005), abdominal aortic aneurysm (Harkin et al. 2005), and acute myocardial infarction (Houck et al. 2005). The increase in same-day mortality risk associated with lower barometric pressure (Schwartz 2000), therefore, may not be mitigated by measures to reduce heat exposure, such as air conditioning. In addition, using more weather variables in estimating the public health risk of hot weather adds more complexity to the task of translating weather forecasts into heat advisories that the public and public officials can understand and respond to.

To reduce the burden on the forecaster, heat health warning systems used in some cities apply a model that uses historic data, SSC, maximum temperature, season, and day in sequence of a heat wave to predict excess mortality (Kalkstein et al. 1996). As noted above, if these predictions are not based on recent data, excess mortality estimates may not be accurate. The substantial unexplained components of variation in daily mortality and sampling error in estimated risk functions add further error to estimates of daily mortality. In addition, local meteorologists still exercise discretion regarding the issuance of heat warnings, apparently considering other criteria, including the forecast heat index (Ebi et al. 2004). Our results show that the forecast maximum heat index and forecast duration of a heat wave are useful measures of the public health risk posed by heat waves in New York City. Heat index may also be simpler to use and apply consistently in issuing heat warnings compared with alternative metrics.

## Conclusions

We found that, among various weather indices, maximum heat index is a useful metric for assessing public health risk due to hot weather in New York City. Although there is no clear threshold for the temperature–mortality relationship during the warm season, the nonlinear relationship found in this analysis supports issuing heat alerts when the maximum heat index is forecast to exceed approximately 95°–100°F, with augmented warnings and response as the forecast heat index and duration of a heat wave increases.

Other jurisdictions should consider conducting similar analyses with recent local data before adopting new heat-alert criteria. Whatever models or metrics are used to estimate public health risk from summertime heat, the benefits of advisories and heat emergency response will, of course, depend on the ability of the public, government agencies, and nongovernmental organizations to take actions that actually reduce heat exposure and health risks.

## Figures and Tables

**Table 1:** Descriptive statistics for daily natural-cause mortality and selected meteorologic parameters, LaGuardia Airport, New York City, May through September, 1997 through 2006.

					Correlations							
Meteorologic factors	Descriptive statistics				Natural-cause deaths	Temperature (°F)				Heat index max (°F)	Dew point temp (°F)	Sea level pressure (mbar)
	Total	Mean ± SD	Min	Max		Min	Avg	Max	Max dev^a^			
Natural-cause deaths	208,380	136.2 ± 14.3	95	248	—							
Temperature (°F)												
Min	—	62.1 ± 11.7	8	86	−0.11	—						
Avg	—	71.1 ± 9.3	32	94	−0.04	0.92	—					
Max	—	79.6 ± 9.1	55	104	0.06	0.59	0.86	—				
Max dev^a^	—	1.0 ± 7.0	−19	25	0.15	0.39	0.63	0.79	—			
Heat index max (°F)	—	80.4 ± 10	55	110	0.07	0.59	0.85	0.99	0.78	—		
Dew point temp (°F)	—	58.3 ± 9.3	27	75	−0.01	0.54	0.66	0.66	0.37	0.70	—	
Sea level pressure (mbar)	—	1015.3 ± 5.6	994	1,034	−0.13	−0.03	−0.07	−0.11	−0.15	−0.13	−0.16	—
Wind speed, mean (miles/hr)	—	9.8 ± 2.8	2	24	0.05	−0.14	−0.22	−0.26	−0.20	−0.24	−0.22	−0.19

Abbreviations: Avg, average; Dev, deviation from normal; Max, maximum; Min, minimum; temp, temperature.

a Daily deviations from average maximum temperature for date recorded at LaGuardia Airport, New York City, 1971–2000.

**Table 2:** Comparison of fit for selected parametric and nonparametric models of the relation of meteorologic metrics and natural-cause mortality.

	Parametric models			Nonparametric models		
Model parameters	Deviance explained (%)	First-order residual auto correlation	Correlation of raw and predicted values on HI ≥ 90°F days	Deviance explained (%)	First-order residual auto correlation	Correlation of raw and predicted values on HI ≥ 90°F days
Long- and short-term temporal trends only	19.8	0.12	0.51	20.0	0.10	0.47
Maximum heat index, lags 0–3	27.6	0.05	0.63	29.7	0.03	0.65
SSC, lags 0–3	27.9	0.05	0.57	28.1	0.04	0.56
Average temperature, lags 0–3	26.5	0.06	0.60	30.7	0.03	0.66
Maximum temperature, lags 0–3	26.3	0.06	0.58	28.5	0.03	0.61
Minimum temperature, lags 0–3	26.2	0.07	0.60	29.0	0.04	0.66
Deviation from normal maximum temperature, lags 0–1	24.6	0.07	0.56	26.3	0.05	0.58
Exponential temperature transformation, lags 0–1	24.7	0.07	0.58	25.0	0.06	0.57
Maximum heat index, lags 0–3 + minimum temperature, lags 0–3 + mean sea level pressure, lags 0–1 + mean wind speed, lags 0–1 + precipitation, lags 0–3	31.0	0.04	0.69	35.6	0.00	0.74
Maximum heat index, lags 0–3 + SSC, lags 0–3	31.7	0.02	0.67	33.2	0.00	0.67

HI, heat index.
